# Evaluation of Retinal Changes in Varicocele Patients With Optical Coherence Tomography Angiography

**DOI:** 10.7759/cureus.20019

**Published:** 2021-11-29

**Authors:** Murat Serkan Songur, Mehmet Caniklioğlu

**Affiliations:** 1 Ophthalmology, Yozgat Bozok University Faculty of Medicine, Yozgat, TUR; 2 Urology, Yozgat Bozok University Faculty of Medicine, Yozgat, TUR

**Keywords:** foveal avascular zone, capillary plexus, vascular density, optical coherence tomography angiography, varicocele

## Abstract

Objective: To evaluate the macula and optic disc in varicocele patients with optical coherence tomography angiography (OCTA).

Methods: The right eyes of 30 patients with varicocele and 30 healthy volunteers were evaluated. Superficial and deep capillary plexus vascular densities, foveal avascular zone (FAZ) width, and optic disc parameters were measured with OCTA.

Results: There was a significant decrease in vascular density in the superficial and deep capillary plexus in the varicocele group compared to the control group, but no difference was found in FAZ and optic disc parameters.

Conclusion: Patients with varicocele have reduced microvascular density in the retina; therefore, it may be beneficial to perform a detailed eye examination with OCTA in these patients.

## Introduction

Varicocele is known as dilatation of plexus pampiniformis and internal spermatic veins and is seen in 10-15% of men. It is the most common cause of infertility in men that can be corrected surgically. Since varicocele is a progressive disorder, its early detection is imperative. While it is seen on the left side with a rate of 90%, in 10% of the cases, it is seen bilaterally [1]. Although varicocele is perceived to cause infertility problems at the age of 18 and above, which is accepted as adult age, it can also be seen in childhood and may cause infertility by creating progressive damage [2]. The fact that the incidence of varicocele is around 53% in the first-degree relatives of male patients with varicocele, which is considerably higher than that of the normal population, indicates that there is a genetic predisposition [3].

Optical coherence tomography angiography (OCTA) is a non-invasive clinical tool that can capture retinal capillary microcirculation at micrometer resolution [4]. It is an imaging method that provides detailed visualization of the retinal vascular network by obtaining and processing the motion contrast of the erythrocytes in the vessel with sequential optical coherence tomography (OCT) scans of a certain retinal area. Unlike fundus fluorescent angiography (FFA), no intravenous contrast agent is used in this angiography method, and therefore the procedure is non-invasive [5].

In this study, we tried to reveal retinal disorders that may be associated with varicocele patients due to genetic or other vascular factors. We applied non-invasive and easily applicable OCTA to patients with varicocele to determine whether dilatation and flow disorders that are seen in the plexus pampiniformis are also present in the retinal microvascular structure. This effect is manifested by a decrease in vascular density in the microvascular structure. We have not come across such a study in the literature.

## Materials and methods

This prospective study was conducted in our hospital's urology and ophthalmology outpatient clinics between March 2021 and May 2021. Informed consent was obtained from all patients and ethical committee approval was obtained from the hospital's ethics committee before initiation of the study (2017-KAEK-189_2021.02.10­_10). We complied with the Helsinki Declaration principles throughout our study.

Clinical varicocele patients were included in the study. The patients were selected among the patients between the ages of 18 and 30 years, who did not have any systemic disease, and who cooperated with OCTA.

All patients underwent a complete and detailed ophthalmologic evaluation including best-corrected visual acuity, intraocular pressure measurement, slit-lamp biomicroscopy, pachymetry, gonioscopy, and three-mirror contact lens fundoscopy. Individuals with uveitis, glaucoma, retinal disease, corneal opacity, high hyperopia and myopia (>6D), patients who could not cooperate during OCTA imaging, and patients with an ocular trauma and surgery history were excluded from the study. Patients with vascular pathologies, epilepsy, diabetes mellitus, and hypertension were also excluded from the study.

In our study, foveal avascular zone (FAZ) width, superficial capillary plexus (SCP) and deep capillary plexus (DCP) vascular vessel density (VD), and vascular densities of the optic disc and peripapillary region were measured with the RTVue XR Avanti OCTA system (Optovue, Inc., Fremont, CA). OCTA images with a quality level of 7 and above were included in the study.

In the examinations performed with OCTA, the DCP and SCP of the retina were evaluated as 6 x 6 mm angioretina. The vascular density of the entire fovea, parafovea, and perifovea was measured in the SCP and DCP. The width of the FAZ, which does not have any vascularization, was evaluated automatically by OCTA.

Peripapillary vascular VD was also measured with OCTA. Peripapillary measurements were made as 4.5 x 4.5 mm in the inner disc, which expresses a 2-mm center of the optic disc and in eight quadrants.

G*Power v.3.1.9.2 (Franz Foul, Universitat Kiel, Germany) software was used for estimated sample size calculation. Based on the independent sample t-test with an alpha value of 0.05, a power of 80%, and an effect size of 0.5, it was found that a minimum of 30 subjects for each group were needed.

Statistical analyses were performed using the SPSS® 22.0 (IBM SPSS Statistics, Armonk, NY) program. We made descriptive statistics of the data. Shapiro-Wilk test was used to evaluate the normality of distribution. We used the chi-square test to compare categorical variables. The Student's t-test was used for pairwise comparison of normally distributed data, while the Mann-Whitney U test was used for non-normally distributed data. A p-value less than 0.05 was considered statistically significant.

## Results

The right eyes of 30 patients diagnosed with varicocele and 30 healthy controls were evaluated in the study. The patients' mean age was 25.20 years in the varicocele group and 24.16 years in the control group (p = 0.369).

In our study, when we looked at the vascular density of the SCP and DCP with OCTA, there was a significant decrease in all quadrants of the fovea and parafovea in the varicocele group compared to the control group. However, in the perifovea, no difference was detected in both the SCP and DCP (Tables 1, 2). Moreover, no statistically significant difference was found between the two groups in FAZ measurements (p = 0.866).

**Table 1:** Superficial capillary plexus values in OCTA imaging of the patients. Group 1: varicocele disease group; Group 2: healthy control group. Continuous data are presented as mean ± standard deviation. OCTA: optical coherence tomography angiography; Inf: inferior; Sup: superior; Nas: nasal; Temp: temporal.

Vascular density	Group 1 (n = 30)	Group 2 (n = 30)	p-value
Whole macula, Inf hemisphere	47.35 ± 4.42, 46.97 ± 4.39	50.11 ± 2.93, 49.96 ± 3.01	0.004, 0.002
Sup hemisphere	47.64 ± 4.76	50.28 ± 2.96	0.029
Fovea	22.02 ± 6.05	27.33 ± 10.57	0.02
Parafovea	49.06 ± 5.50	52.63 ± 4.80	0.006
Parafovea Temp	48.51 ± 6.69	52.77 ± 4.01	0.004
Parafovea Sup	49.89 ± 6.23	53.11 ± 5.03	0.026
Parafovea Nas	48.20 ± 5.46	51.71 ± 5.29	0.009
Parafovea Inf	49.62 ± 5.66	52.95 ± 5.65	0.011
Perifovea	48.96 ± 5.06	51.11 ± 2.93	0.093
Perifovea Temp	44.91 ± 5.14	47.32 ± 2.79	0.067
Perifovea Sup	48.45 ± 4.22	51.25 ± 2.34	0.079
Perifovea Nas	52.28 ± 3.91	54.90 ± 2.81	0.29
Perifovea Inf	48.96 ± 5.06	51.11 ± 2.93	0.049

**Table 2:** Deep capillary plexus values in OCTA imaging of the patients. Group 1: varicocele disease group; Group 2: healthy control group. Continuous data are presented as mean ± standard deviation. OCTA: optical coherence tomography angiography; Inf: inferior; Sup: superior; Nas: nasal; Temp: temporal.

Vascular density	Group 1 (n = 30)	Group 2 (n = 30)	p-value
Whole macula	47.08 ± 6.17	50.99 ± 4.90	0.009
Sup hemisphere, Inf hemisphere	46.28 ± 6.12, 47.82 ± 6.77	50.60 ± 5.11, 51.06 ± 5.38	0.004, 0.045
Fovea	33.14 ± 7.42	38.01 ± 6.67	0.010
Parafovea	51.83 ± 5.38	55.50 ± 3.79	0.013
Parafovea Temp	52.46 ± 6.91	55.65 ± 4.03	0.033
Parafovea Sup	51.25 ± 6.56	54.69 ± 3.97	0.017
Parafovea Nas	54.38 ± 4.88	56.91 ± 3.98	0.032
Parafovea Inf	50.62 ± 5.91	54.43 ± 4.41	0.006
Perifovea	48.93 ± 6.83	51.45 ± 4.15	0.090
Perifovea Temp	51.13 ± 6.54	53.83 ± 3.93	0.058
Perifovea Sup	46.89 ± 7.21	50.02 ± 4.97	0.055
Perifovea Nas	47.96 ± 7.71	50.16 ± 4.69	0.188
Perifovea Inf	49.72 ± 7.79	51.32 ± 5.55	0.364

In our study, no statistically significant difference was found between the two groups in 4.5 mm angiodisc images of the entire optic disc, inner disc, peripapillary region, and eight quadrants (Table 3).

**Table 3:** Peripapillary vascular density values. Group 1: varicocele disease group; Group 2: healthy control group. Continuous data are presented as mean ± standard deviation. Inf: inferior; Sup: superior; Nas: nasal; Temp: temporal.

	Group 1 (n = 30)	Group 2 (n = 30)	p-value
Whole	49.05 ± 2.51	49.70 ± 2.77	0.340
Inside disc	48.75 ± 5.37	49.95 ± 4.11	0.615
Peripapillary	51.62 ± 2.71	51.83 ± 3.10	0.781
Sup hemisphere	51.82 ± 3.26	52.56 ± 3.68	0.410
Inf hemisphere	51.39 ± 2.35	51.71 ± 3.48	0.682
Nasal sup	49.22 ± 3.09	50.44 ± 4.62	0.235
Nasal inf	48.39 ± 4.42	48.42 ± 4.89	0.976
Inferior nasal	50.84 ± 4.57	49.32 ± 5.80	0.264
Inferior temporal	57.21 ± 4.15	57.36 ± 4.75	0.897
Temporal inferior	51.76 ± 3.72	53.27 ± 4.07	0.139
Temporal superior	53.96 ± 5.60	55.27 ± 3.44	0.282
Superior temporal	54.54 ± 4.05	55.23 ± 5.10	0.562
Superior nasal	49.44 ± 5.61	50.11 ± 5.36	0.790

## Discussion

In this study, we evaluated the microvascular structure of the retina with OCTA and found a significant decrease in vascular density in the fovea and parafovea regions of the DCP and SCP in varicocele patients compared to healthy controls. However, there was no statistically significant difference between the two groups for FAZ and optic disc parameters.

Varicocele is the most common cause of infertility in men [6]. There are different theories about factors that can increase pressure in the testicular veins in the varicocele. The first of these may be enlargement of the venous structures in the testicles due to compression of the testicular vein between the aorta and superior mesenteric artery. In addition, the lack of an adequate valve system in the testicular veins causes blood to escape into the testicular venous structures and increase the pressure. The other theory is that the right testicular vein enters obliquely the inferior vena cava, while the left testicular vein opens directly into the left renal vein. All these features cause dilatation and tortuosity by causing an increase in venous pressure in testicular veins [7]. Yet another theory is that in cases with varicocele, high concentrations of reflux of metabolites such as catecholamines and prostaglandin E and F from the kidney and adrenal glands may occur [8]. Leydig cell function is also affected in varicocele. Possible underlying mechanisms in this regard include venous stasis, oxidative stress, and increased testicular temperature [9].

In one of the few ophthalmological studies on varicocele, Kumanov et al. found that the varicocele prevalence in light-eyed men was statistically significantly lower than in dark-eyed men. However, no relationship was found between semen parameters and eye color. They suggested that there may be a genetic link [10].

Because the retina's microvascular system, arterioles, and venules can be evaluated non-invasively, these vascular changes in the retina may serve as markers of the preclinical situation of systemic microvascular disease [11]. Coskun et al. compared retinal microvascular diameters of varicocele patients with a healthy control group using a digital retinal camera. They found that the central retinal venular equivalent (CRVE) and arterio-venular ratio (AVR) were significantly different in varicocele patients compared to the control group. There was no difference between the groups in terms of central retinal arteriolar equivalent (CRAE) [12]. This study is an indication that the venous problems seen in varicocele may also be in the retina. Similarly, in our study, we found a decrease in vascular density in both the SCP and DCP, fovea, and parafovea regions. However, the absence of such a decrease in the perifovea might be due to the presence of larger vessels in this region that can shadow the capillaries.

Our results, as results from some previous studies, support the idea that varicocele is a systemic disorder [13,14]. In this study, we determined that there are simultaneous changes in the microvascular structure of the retina in varicocele patients. The cause of these retinal changes may be due to three main mechanisms. The first is venous structural changes, venous insufficiency, and hypoxia. The second is impaired antioxidant defense, while the third, is the effect of genetic factors.

Lee et al. have shown that there was hypoxia in the testes of patients with varicocele [15]. In addition, it has been reported that a decrease in vasa vasorum increases hypoxia [16]. Hypoxia-induced release of many vasoactive chemicals from tissues, especially vascular endothelial growth factor (VGEF), may impact the retinal microvascular structure. Likewise, nitric oxide (NO) is very effective in regulating ocular circulation and retinal vascular tone [17]. The potential of metabolic and systemic conditions to alter NO production in varicocele may affect the calibration of the retinal vasculature [18].

Inflammation may be systemic in the seminal region or subclinically. Both pro-inflammatory cytokines and other inflammatory mediators play a role in this event [19]. This inflammation also reduces the systemic antioxidant defense and leads to the involvement of retinal veins [20].

Our study has some limitations as it is the first study on this subject and the sample group is limited. However, the retinal microvascular changes revealed in this study require further investigation.

## Conclusions

Considering that varicocele is a very common disorder seen in men and that these patients have decreased vascular density in the macula in OCTA images, we think that a detailed ophthalmological examination together with OCTA would be beneficial after diagnosing these patients with varicocele. More experimental research and extensive clinical studies are needed to confirm our results.
